# Correlação entre a Circunferência de Cintura e Medidas Centrais da Pressão Arterial

**DOI:** 10.36660/abc.20210432

**Published:** 2022-05-24

**Authors:** Gilberto Campos Guimarães, Lucas Tavares Silva, Ruth Mellina Castro e Silva

**Affiliations:** 1 Universidade Federal de Goiás Rio Verde GO Brasil Universidade Federal de Goiás, Rio Verde, GO – Brasil; 2 Universidade Federal de Jataí – Medicina Jataí GO Brasil Universidade Federal de Jataí – Medicina, Jataí, GO – Brasil

**Keywords:** Doenças Cardiovasculares, Aterosclerose, Pressão Arterial, Rigidez Vascular, Circunferência da Cintura, Análise de Onda de Pulso, Avaliação de Resultados em Cuidados de Saúde

## Abstract

**Fundamento:**

A rigidez arterial é um forte preditor de doença cardiovascular (DCV). Medidas de gordura corporal, como a circunferência da cintura (CC), têm sido associadas à DCV na idade adulta.

**Objetivos:**

O objetivo deste estudo foi avaliar a associação da rigidez arterial, medida por tonometria de aplanação-Sphygmocor, com a CC.

**Métodos:**

Estudo observacional com 240 participantes que fazem consultas de rotina no ambulatório de clínica médica de um hospital universitário. Os participantes foram entrevistados e tiveram as medidas centrais da pressão arterial (MCPA), parâmetros antropométricos, gordura abdominal e gordura visceral mensurados. Foram u tilizados os testes *t* pareado e não pareado e qui-quadrado. Foi a dotado nível de significância de 5%.

**Resultados:**

Dos 240 participantes, 51,82% era do sexo masculino com idade média de 59,71(±14,81) anos e CC média de 99,87 (±11,54) cm. Os valores médios das MCPA foram: Pressão arterial central (PAC) = 130,23 (91-223) mmHg, velocidade de onda de pulso (VOP) = 9,8 (5,28-19,6)m/s e *Augmentation Index* [Índice de amplificação (AI)] = 29,45 (-14-60). A VOP e a PAC foram altamente correlacionadas com uma CC com p<0,001 e p=0,02, respectivamente; porém, a mesma correlação positiva não foi encontrada entre a CC e o AI (p=0,06).

**Conclusão:**

O presente estudo mostrou uma associação positiva entre a CC e a rigidez arterial, através da velocidade de onda de pulso carotídeo femoral (VOP-cf) e o AI, sendo mais forte com a VOP-cf, sugerindo a avaliação do efeito da CC na saúde vascular como método de auxílio no tratamento precoce das DCV e na prevenção de desfechos clínicos.

## Introdução

A doença cardiovascular (DCV) é a principal causa de morte no Brasil e no mundo, determinando aumento da morbidade e incapacidade ajustadas pelos anos de vida.^1^ Sua prevalência, cada vez mais elevada, tem sido reflexo do envelhecimento e adoecimento da população, mesmo após otimização de políticas públicas de prevenção.^1^

A presença dos fatores de risco clássicos (hipertensão, dislipidemia, obesidade, sedentarismo, tabagismo, diabetes e histórico familiar) aumenta a probabilidade pré-teste de DCV – com ênfase para a doença arterial coronariana (DAC) – e norteia a prevenção primária e secundária.^2^

A obesidade está associada a um aumento na incidência de insuficiência cardíaca (IC), infarto do miocárdio (IM), acidente vascular cerebral e morte.^3,4^ Estudos de pacientes com sobrepeso e obesos com DCV sugerem um “paradoxo da obesidade”, pelo qual o índice de massa corporal (IMC) elevado pode estar associado a menor mortalidade e eventos cardiovasculares.^5,6^

Por outro lado, o índice de massa corporal (IMC) é incapaz de diferenciar a massa magra da gordura^7^e tem sido proposto o uso de outras medidas de adiposidade, como circunferência da cintura (CC), que se mostrou um bom preditor de gordura abdominal e risco cardiovascular.^8,9^

Parte do processo aterosclerótico está relacionado ao aumento da rigidez arterial, que tem como principal biomarcador a velocidade de onda de pulso (VOP).^10,11^ A rigidez arterial é um importante preditor independente de mortalidade cardiovascular em diversas populações de pacientes, incluindo pacientes hipertensos.^12–14^

Consistente com o papel central da rigidez arterial na função cardiovascular, as medidas da rigidez arterial, por predizerem risco cardiovascular, podem representar um biomarcador promissor na prevenção de desfechos cardiovasculares.^15^

Com base no conhecimento atual da significância das medidas da rigidez arterial no prognóstico de doenças cardiovasculares, o presente estudo visa analisar a associação da CC com o perfil hemodinâmico central, possibilitando correlacionar a identificação precoce dos pacientes que estão expostos ao maior risco cardiovascular, para implementar mudanças no estilo de vida e tratamentos que podem evitar complicações e progressão das doenças cardiovasculares. Conduzimos o presente estudo com o objetivo de avaliar a associação entre os valores da CC com as medidas centrais da pressão arterial (MCPA) - VOP, *Augmentation Index* (AI) e pressão arterial central (PAC).

## Métodos

### Desenho do estudo e participantes

Trata-se de um estudo observacional transversal. Os participantes elegíveis eram aqueles atendidos no ambulatório de clínica médica de um hospital universitário, composto de um laboratório de referência em envelhecimento vascular, onde se avalia a rigidez arterial por tonometria de aplanação com medidas da velocidade de onda de pulso (SphygmoCor®). Esse é um instrumento que fornece medição da velocidade de onda de pulso carotídeo-femoral (VOP-cf) nas artérias femoral e carótida por tonometria de aplanação. O sistema, validado e utilizado há décadas, é atualmente considerado o método não invasivo padrão-ouro para aquisição de medidas hemodinâmicas centrais.^16^

Adotou-se como critério de inclusão participantes com idade maior a 18 anos. Os critérios de exclusão utilizados foram: ausência de técnicas adequadas para verificar a PA periférica,^17^ medidas de PA periférica não realizadas em aparelho digitais, calibrados e validados; participação em outros protocolos de pesquisa por menos de um ano conforme regulamentação da ANVISA – Brasil; doenças crônicas em estágios terminais; doença cardiovascular prévia, incluindo doença arterial coronariana (IM, angina, cirurgia de revascularização anterior ou angioplastia) ou acidente vascular cerebral (acidente vascular cerebral isquêmico ou AIT) por <6 meses. Os critérios de exclusão para DCV prévia apresentados foram definidos a partir de informações obtidas dos participantes por entrevista direta ou evidências por meio de exames complementares.

Na unidade ambulatorial citada são atendidos em média 40 pacientes por dia, com média de 200 pacientes por semana, com realização de medidas centrais de pressão arterial (MCPA) nos pacientes indicados. A seleção dos participantes foi mediante convite para participação àqueles que atendiam aos critérios de inclusão e exclusão, e aceite do paciente. O tamanho da amostra foi de 247 participantes, conforme conveniência do campo.

### Dados coletados

A coleta de dados foi realizada no momento do atendimento de rotina do paciente no ambulatório nos meses de junho e outubro de 2019. Foram coletadas informações como gênero, idade e comorbidades associadas avaliadas pela autorreferência e através das medicações de uso crônico. Foi considerado tabagista aquele com o consumo de pelo menos um cigarro por dia.^18^

Foram coletados ainda peso (em kg) e altura (em m) com o cálculo do índice de massa corporal (fórmula de Quetelet);^19^ e circunferência de cintura (em cm). Todas as medidas foram aferidas com indivíduos na posição de pé usando os padrões criados para estudos de saúde populacional.^19,20^

A pesquisa de dano cardíaco e vascular de órgão alvo foi realizada através do ecodopplercardiograma e doppler de carótidas, utilizando um aparelho modelo TOSHIBA Xsario. Foram analisados os seguintes parâmetros: medidas do septo interventricular e da parede posterior do ventrículo esquerdo, índice de massa ventricular esquerda e volume do átrio esquerdo, no Ecodopplercardiograma, e medida da espessura da íntima média e presença de placas carotídeas, no doppler de carótidas

Já a Microalbuminúria foi definida como excreção de albumina na urina entre 30 e 300 mg / 24 horas^21^ realizada por meio de coleta de urina de 24h ou na presença do exame com menos de 6 meses de realização.

A VOP-cf foi medida com o dispositivo CvMS Sphygmocor (versão 9 do software, AtCor Medical), por tonometria de aplanação (PWVton) sequencialmente na artéria carótida e femoral, bloqueado por um sinal de eletrocadiograma gravado simultaneamente.^22^

A mensuração da PAC foi realizada por tonometria de aplanação, em aparelho SphygmoCor®, calibrado e validado clinicamente pela *European Society of Hypertension* (ESH) e pela *European Society Cardiology* (ESC).^23^ O instrumento consiste em um tonômetro (sensor ou transdutor portátil de pressão) acoplado a um computador com software dedicado para coleta e análise dos dados. Quando usado na artéria radial, o SphygmoCor® também obtém medidas relacionadas à pressão arterial sistólica central (PASC) e diastólica (PADC), amplificação da pressão de pulso (PPA), pressão de pulso central (PPC) e *Augmentation Index* (AI x) por função de transferência. Quando usado nas artérias carótida e femoral, o sistema também calcula a VOP.

### Tamanho amostral

Foi realizado cálculo amostral para estimativa de prevalência em uma população finita de 1250 indivíduos, prevalência de hipertensão sistólica e diastólica central de 13,7%>^24^, erro absoluto tolerável de 5%, e coeficiente de confiança de 97,5%, totalizando uma amostra de 200 pacientes. Adicionou-se 20% para garantia de perdas por deficiências no preenchimento adequado do questionário.

Ao final da coleta foram obtidos dados de 247 participantes e excluídos 7, dos quais 5 por ausência de dados sobre CC e outros dois por mais de 30% do questionário incompleto, findando com uma amostra de 240 pacientes.

### Análise estatística

Os dados categóricos estão apresentados em frequências absolutas (n) e relativas (%). As variáveis numéricas estão apresentadas em média e desvio-padrão da média ou mediana e intervalo interquartil (percentil 25-75). Para verificar a normalidade da distribuição dos dados, utilizou-se o teste de Shapiro Wilk. Para comparação entre grupos, foi utilizado o teste U de Mann Whitney ou teste de Kruskal Wallis ou teste t-Student não pareado ou ANOVA one-way. Para a análise de correlação entre variáveis, calculou-se o coeficiente de correlação de Spearman ou de Pearson.

Foi ainda realizada análise de regressão linear e logística tendo como desfechos os exames cardiológicos e a variável determinante a CC classificada em alterada e normal; as demais variáveis foram usadas como ajustes para determinação do potencial confundidor. As análises foram realizadas no STATA versão 14.p e para todos os testes considerou-se o nível de significância de 5%.

### Aspectos éticos

O projeto de pesquisa foi avaliado e aprovado pelo Comitê de Ética em Pesquisa do Hospital das Clínicas da Universidade Federal de Goiás (UFG), número do parecer: 3.907.884, com assinatura do Termo de Consentimento Livre e Esclarecido (TCLE) por parte de todos os participantes.

## Resultados

Participaram do estudo 240 pacientes atendidos no ambulatório de clínica médica de um hospital universitário; entretanto, não foi possível coletar algumas informações de todos os pacientes. A amostra foi constituída em sua maioria do sexo masculino, com meia idade, sobrepeso e média da CC acima do limite superior da normalidade para o sexo feminino.^17^ Verificou-se elevada prevalência de tabagistas^25^ e mais de um quarto da população estudada com DCV (Tabela 1). As MCPA mostraram uma média de VOP próxima do limite superior da normalidade para lesão de órgão alvo (VOP > 10m/s)^17,23^ e de PAC acima do limite superior da normalidade para a população estudada^17^ (Tabela 1).

**Tabela 1:** Caracterização da amostra e relação com classificação da circunferência da cintura de pacientes atendidos no ambulatório de clínica médica de um hospital universitário^17^

	Amostra total n=240	Circunferência da Cintura		p-valor
		Normal 34(14,17%)	Alterada 206(85,83%)	
Idade, anos, mediana [IIQ] , n=240	60,25 [51,50-70,00]	54,00 [38,00-64,00]	63,00 [53,00-71,00]	0,004^1^
Sexo, n(%), n=240				0,001^3^
Feminino	115(47,92)	7(20,59)	108(52,43)	
Masculino	125(52,08)	27(79,41)	98(47,57)	
Peso, kg, mediana [IIQ], n=237	75,10 [67,00;84,00]	64,80 [55,80-76,70]	75,75 [69,55-85,10]	<0,001^1^
Índice de massa corporal, kg/m^2^, mediana [IIQ] , n=238	27,98 [25,40-31,86]	22,94 [21,47-25,40]	28,62 [26,23-32,46]	<0,001^1^
Estado Nutricional, n(%), n=238				<0,001^3^
Eutrófico	72(30,25)	24(72,73)	48(23,41)	
Excesso de peso	166(69,75)	9(27,27)	157(76,59)	
Circunferência da cintura, cm, média (DP) , n=240	99,95(11,59)	84,52(7,83)	102,49(10,04)	<0,001^2^
Hábito tabagista, n(%), n=240				0,023^4^
Nunca Fumante	166(69,17)	24(70,59)	142(68,93)	
Fumante	37(15,42)	9(26,47)	28(13,59)	
Ex-fumante	37(15,42)	1(2,94)	36(17,48)	
Doenças crônicas, n(%), n=240				
Hipertensão arterial	213(88,75)	28(82,35)	185(89,81)	0,203^3^
Dislipidemia	179(74,58)	19(55,88)	160(77,67)	0,007^3^
Acidente vascular encefálico	46(19,17)	5(14,71)	41(19,90)	0,639^4^
Diabetes Mellitus	95(39,58)	6(17,65)	89(43,20)	0,005^3^
Microalbuminúria, n(%), n=212	81(38,21)	7(26,92)	74(39,78)	0,282^4^
PASP,mmHg, mediana [IIQ], n=240	140,00 [128,00-154,00]	132,00 [122,00-154,00]	140,00 [129,00-154,00]	0,068^1^
PADP,mmHg, mediana [IIQ], n=240	77,50 [70,00-86,00]	77,50 [70-84]	77,50 [70,00-87,00]	0,464^1^
VOP, m/s, mediana [IIQ], n=239	9,30 [7,90-11,30]	8,91 [7,28-10,32]	9,41 [8,00-11,40]	0,151^1^
PAC,mmHg, mediana [IIQ], n=240	128,00 [116,00-141,00]	124,00 [110,00-138,00]	129,00 [117,00-141,00]	0,106^1^
AI x, %, média (DP), n=240	29,55(12,46)	28,93(15,29)	29,65(11,96)	0,756^2^
Dano cardíaco, n(%), n=183	98(53,55)	7(31,82)	91(56,52)	0,029^3^
Dano vascular, n(%), n=112	87(77,68)	9(69,23)	78(78,79)	0,482^4^

IIQ: intervalo interquartil; DP: desvio-padrão; n: frequência absoluta; % frequência relativa; PCR: proteína C-reativa; PASP: pressão arterial sistólica periférica; PADP: pressão arterial diastólica periférica; PASC: pressão arterial sistólica central; AIx: Augmentation index; VOP: velocidade da onda de pulso.

1 Mann-Whitney;

2 teste de t-Student para amostras independentes;

3 Teste de Qui-quadrado;

4 Teste exato de Fisher, todos com 5% de significância.

Nos indivíduos do sexo feminino, excesso de peso, fumantes e ex-fumantes, dislipidemia, diabetes mellitus e dano cardíaco, houve maior frequência de CC alterada. As medianas de idade, peso e IMC, são maiores nos indivíduos com CC alterada do que naqueles com CC eutrófica (Tabela 1).

Foram observados menores valores de AIx e maiores valores de VOP no sexo masculino. Houve também maior VOP e PAC nos pacientes com dano vascular, mas nenhuma diferença nesses parâmetros foi encontrada com relação ao hábito tabagista (Tabela 2).

**Tabela 2:** Diferenças de MCPA entre sexo, Doppler de carótidas e hábito tabagista

Variáveis	VOP		PAC		AIx	
	Mediana [IQ]	p-valor	Mediana [IQ]	p-valor	Média (DP)	p-valor
Sexo		<0,001^1^		0,526^1^		0,010^3^
Feminino	8,88 [7,68-10,10]		127,00 [114,00-140,00]		31,70(12,01)	
Masculino	10,10 [8,30-12,15]		129,00 [117,00-141,00]		27,58(12,58)	
Doppler de carótidas alterado		0,004^1^		0,037^1^		0,072^3^
Não	8,90 [7,70;10,20]		127,00 [114,00;135,00]		26,32(13,66)	
Sim	10,36 [9,20-12,10]		133,00 [119,00-148,00]		30,93(10,38)	
Hábito tabagista		0,219^2^		0,682^2^		0,437^4^
Nunca Fumante	9,21 [7,84-11,36]		128,50 [116,00-146,00]		30,20(12,61)	
Fumante	9,11 [7,93-10,61]		128,00 [112,00-138,00]		27,45(9,94)	
Ex-fumante	10,10 [8,70-11,50]		126,00 [116,00-136,00]		28,73(13,97)	

n: frequência absoluta de indivíduos; IIQ: Intervalo-interquartil; DP: desvio padrão da média; VOP: Velocidade de onda de pulso; PAC: Pressão arterial central; AIx: Augmentation index; p-valor obtido por

1 Teste de Mann-Whitney, ou

2 Teste de Kruskal-Wallis;

3 Teste t-Student;

4 teste de ANOVA oneway, todos com 5% de nível se significância.

Observou-se correlação inversa e significativa entre AIx e Peso, IMC e CC. Verificou-se também uma correlação direta e significativa entre: VOP e idade e CC; entre PAC e idade e CC; e entre AIx e idade (Tabela 3).

**Tabela 3:** Correlação entre CC e MCPA

Variáveis	Correlação Coeficiente de correlação rho (p-valor)		
	VOP^1^	PAC^1^	AIx^2^
Idade (anos)	0,54 (<0,001)	0,20 (0,002)	0,33 (<0,001)
Peso (kg)	0,09 (0,192)	0,03 (0,671)	-0,31 (<0,001)
IMC (kg/m^2^)	0,11 (0,095)	0,11 (0,080)	-0,17 (0,009)
CC (cm)	0,33 (<0,001)	0,15 (0,020)	-0,10 (0,131)

MCPA: Medidas Centrais da Pressão Arterial; VOP: Velocidade de onda de pulso; PAC: Pressão arterial central; AIx: Augmentation index; IMC: Índice de Massa Corporal; CC: Circunferência de Cintura;.

1 Teste de correlação de Spearman ou

2 de Pearson, com 5% de nível de significância.

Na análise bruta de associação, foi verificada associação direta entre a CC alterada e a PASP. Ao utilizar um modelo ajustado para idade e sexo, houve associação inversa da CC alterada com o AI x. Em outro modelo ajustado por idade, sexo, hábito tabagista, estado nutricional e comorbidades, a CC alterada não esteve associada a nenhum dos parâmetros avaliados. Por fim, no modelo ajustado por variáveis determinantes, a CC alterada foi determinante apenas para a PASP (Tabela 4).

**Tabela 4:** Associação entre Circunferência da cintura e Exames cardiológicos

	Modelo 1			Modelo 2			Modelo 3			Modelo 4		
	Coef.	IC_95%_	p	Coef.	IC_95%_	p	Coef.	IC_95%_	p	Coef.	IC_95%_	p
PASP*	0,02	0,00;0,05	0,042	0,02	-0,00;0,05	0,074	0,01	-0,02;0,04	0,495	0,03	0,00;0,06	0,030
PADP*	0,01	-0,01;0,04	0,270	0,02	0,00;0,05	0,046	0,01	-0,02;0,03	0,594	0,00	-0,03;0,03	0,812
VOP*	0,03	-0,01;0,07	0,120	0,00	-0,03;0,04	0,792	-0,00	-0,04;0,04	0,867	-0,01	-0,05;0,03	0,705
PAC*	0,02	-0,00;0,04	0,084	0,01	-0,01;0,04	0,321	0,00	-0,03;0,03	0,905	0,02	-0,01;0,05	0,180
AI x	0,72	-3,83;5,27	0,756	-4,47	-8,90-0,04	0,048	-2,87	-7,91;2,14	0,259	2,65	-2,31;7,62	0,294
Dano cardíaco	1,02	0,07;1,97	0,034	0,87	-0,21;1,96	0,115	0,09	-1,14;1,33	0,882	-0,08	-1,51;1,36	0,916
MAPA alterado	-0,50	-1,71;0,70	0,415	-0,24	-1,49;1,01	0,706	-0,93	-2,42;0,55	0,218	-1,55	-3,13;0,03	0,054
Dano vascular	0,50	-0,77;1,77	0,440	1,18	-0,32;2,69	0,124	0,30	-1,50;2,10	0,743	1,08	-0,87;3,03	0,279

Coef.: Coeficiente da regressão linear ou logística; IC95%: intervalo de confiança de 95%; VOP: Velocidade de onda de pulso; PAC: Pressão arterial central; AIx: Augmentation index; MAPA: Monitoramento ambulatorial da pressão arterial.

1 Análise de regressão linear

2 Análise de regressão logística, usando como variável determinante circunferência da cintura classificada em normal vs alterada e variáveis dependentes dos exames cardiológicos.

* Usada variável na escala logarítmica devido à ausência da normalidade. Modelo 1 – bruto; Modelo 2 - ajustado por idade e sexo; Modelo 3 – ajustado por idade, sexo, hábito tabagista, estado nutricional e comorbidades; Modelo 4 – ajustado por variáveis com p<0,20 na análise binária.

## Discussão

O excesso de obesidade abdominal está associado a uma variedade de anormalidades metabólicas e DCV.^8,26^ A medida da CC é empregada como indicador substituto de obesidade visceral para predizer morbimortalidade em nível populacional,^27–29^ além de ser um biomarcador de baixo custo e fácil manuseio.^30^

A rigidez arterial também está relacionada à DCV e à aterosclerose^31^e tem sido um forte preditor independente de eventos coronários e mortalidade cardiovascular em vários grupos de pacientes.^12,32^ Neste estudo, examinamos as relações entre rigidez arterial medida por VOP e um fator de risco cardiovascular em específico: CC de 240 participantes.

As associações univariadas foram, portanto, significativas entre CC e todos os componentes da MCPA, à exceção do AIx. A correlação mais forte foi observada entre a CC e a VOP-cf (p<0,001), o que não é surpreendente, considerando que, atualmente, é o método padrão-ouro na avaliação da rigidez arterial.^33^

Em nosso estudo, a circunferência da cintura foi um determinante significativo de rigidez arterial através da VOP-cf. A associação entre aumento da gordura corporal e alta rigidez arterial também foi encontrada em outros estudos observacionais, transversais e longitudinais, em concordância com nossos achados.^34–36^ Outros mecanismos, como os que envolvem as adipocinas e a regulação endotelial também podem explicar essa associação,^35^ assim como a hipótese de um impacto negativo na saúde de grandes artérias causado pela adiposidade abdominal.^37^

Choi et al.,^38^ mostraram ausência de correlação significativa entre CC e VOP em seu estudo,^38^ podendo ser explicada pelo fato de que a CC não consegue distinguir entre gorduras viscerais e subcutâneas.^39^

Trabalhos transversais anteriores demonstram que o aumento da obesidade abdominal estava associado à diminuição do AIx. Esse achado pode ter sido devido a uma diminuição no gradiente de pressão aórtica transmural e consequente redução do ponto de rigidez operacional da aorta ou à disfunção ventricular esquerda subclínica que se manifesta como um menor grau de aumento de pressão para qualquer magnitude de reflexão dada.^40,41^

Nosso estudo também apresentou ausência de correlação da CC com o AIx, porém com p limítrofe (p=0,06), sugerindo que uma provável amostra maior poderia mostrar um resultado diferente. Quando ajustado para idade, houve também uma associação inversa entre CC e AIx.

Shiva et al, no entanto, que avaliaram as mudanças no AIx ao longo de um período de aproximadamente 3 anos, descobriram que o aumento da circunferência abdominal ao longo do tempo estava associado a um aumento no AIx. A relação direta prospectiva entre obesidade abdominal e AIx sugere uma disfunção vascular progressiva causada pela obesidade, resultando em aumento tardio da pressão sistólica.^42^

Nosso estudo revelou que o aumento da CC tinha uma associação positiva (p=0,002) com o aumento da PAC. Uma coorte representativa de 2742 adultos em Taiwan apresentou uma análise multivariada, que revelou que a CC maior foi, independentemente, associada à elevada PAC.^43^ A mesma associação também foi encontrada em adolescentes na cidade de Salvador, Brasil.^44^ Estudos anteriores apresentaram resultados semelhantes ao nosso,^24,45^ sugerindo o benefício da medida da PAC como melhor abordagem na patogênese das doenças cardiovasculares.

Nossos resultados devem ser interpretados dentro do contexto das limitações potenciais do estudo. Em primeiro lugar, a maioria dos participantes do nosso estudo tinha pelo menos um fator de risco CV entre hipertensão, dislipidemia, diabetes, excesso de peso ou comorbidades cardiovasculares. Embora esses fatores tenham sido devidamente contabilizados, nossos dados podem não ser representativos de toda uma população.

Por fim, estima-se que a presença de um volume amostral mais expressivo possa melhorar o poder estatístico do trabalho e reforçar os benefícios dos resultados apresentados.

## Conclusão

Este estudo demonstrou uma correlação positiva entre a CC e a Rigidez arterial medida pela VOP-cf e PAC, sugerindo a avaliação do efeito da CC na saúde vascular como método de auxílio no tratamento precoce das DCV e na prevenção de desfechos clínicos. Portanto, estudos futuros para determinar a relação entre obesidade abdominal e o risco de rigidez arterial podem considerar a CC para estimar com maior precisão. Nosso estudo fornece informações que requerem confirmação por um ensaio clínico randomizado, em grande escala, porque os efeitos dos estudos observacionais podem ser superestimados.
