# Supplementary material for: Correlação entre a Circunferência de Cintura e Medidas Centrais da Pressão Arterial
Source: Arq Bras Cardiol. 2022 May 24;119(2):257–64. [Article in Portuguese] doi: 10.36660/abc.20210432 (PMC9363063; doi:10.36660/abc.20210432)
Supplement: Translation

# Translation: Correlation among Waist Circumference and Central Measures of Blood Pressure

**Authors:** Gilberto Campos Guimarães, Lucas Tavares Silva, Ruth Mellina Castro e Silva

**Affiliations:** 1 Rio Verde GO Brazil Universidade Federal de Goiás, Rio Verde, GO – Brazil; 2 Jataí GO Brazil Universidade Federal de Jataí – Medicina, Jataí, GO – Brazil

**Keywords:** Cardiovascular Diseases, Atherosclerosis, Blood Pressure, Vascular Stiffness, Waist Circumference, Pulse Wave Analysis, Outcome Assessment, Health Care

## Abstract

**Background:**

Arterial stiffness is a strong predictor of cardiovascular disease (CVD). Body fat measures such as waist circumference (WC) have been associated with CVD in adulthood.

**Objectives:**

The objective of this study was to evaluate the association of arterial stiffness, measured by applanation tonometry-Sphygmocor, with WC.

**Methods:**

Observational study with 240 participants who make routine consultations at the outpatient clinic of a university hospital. Participants were interviewed and had central blood pressure measurements (CBPM), anthropometric parameters, abdominal fat and visceral fat measured. Paired and unpaired t and chi-square tests were used. A significance level of 5% was adopted.

**Results:**

Of the 240 participants, 51.82% were male with a mean age of 59.71(±14.81) years and a mean WC of 99.87 (11.54) cm. Mean CBPM values were: Central arterial pressure (CAP) = 130.23 (91-223) mmHg, pulse wave velocity (PWV) = 9.8 (5.28-19.6)m/s and Augmentation Index [Amplification Index (AI)] = 29.45 (-14-60). PWV and CAP were highly correlated with WC with p<0.001 and p=0.02, respectively; however, the same positive correlation was not found between WC and AI (p=0.06).

**Conclusion:**

The present study showed a positive association between WC and arterial stiffness, through the femoral carotid pulse wave velocity (cf-PWV) and AI, being stronger with cf-PWV, suggesting the evaluation of the effect of WC in vascular health as a method of aid in the early treatment of CVD and in the prevention of clinical outcomes.

## Introduction

Cardiovascular disease (CVD) is the leading cause of death in Brazil and in the world, determining an increase in morbidity and disability adjusted for years of life.^1^ Its increasingly high prevalence has been a reflection of the aging and illness of the population, even after optimizing public prevention policies.^1^

The presence of classic risk factors (hypertension, dyslipidemia, obesity, physical inactivity, smoking, diabetes and family history) increases the pre-test probability of CVD – with emphasis on coronary artery disease (CAD) – and guides primary and secondary prevention.^2^

Obesity is associated with an increased incidence of heart failure (HF), myocardial infarction (MI), stroke and death.^3,4^ Studies of overweight and obese patients with CVD suggest an “obesity paradox”, whereby high body mass index (BMI) may be associated with lower mortality and cardiovascular events.^5,6^

On the other hand, the body mass index (BMI) is unable to differentiate lean mass from fat^7^ and the use of other measures of adiposity, such as waist circumference (WC), has been proposed to be a good predictor of abdominal fat and cardiovascular risk.^8,9^

Part of the atherosclerotic process is related to increased arterial stiffness, whose main biomarker is pulse wave velocity (PWV).^10,11^ Arterial stiffness is an important independent predictor of cardiovascular mortality in diverse patient populations, including hypertensive patients.^12–14^

Consistent with the central role of arterial stiffness in cardiovascular function, arterial stiffness measurements may represent a promising biomarker in the prevention of cardiovascular outcomes, as they predict cardiovascular risk.^15^

Based on the current knowledge of the significance of arterial stiffness measures in the prognosis of cardiovascular diseases, the present study aims to analyze the association of WC with the central hemodynamic profile, making it possible to correlate the early identification of patients who are exposed to greater cardiovascular risk, to implement lifestyle changes and treatments that can prevent complications and progression of cardiovascular disease. We conducted the present study with the aim of evaluating the association between WC values and central blood pressure measurements (CBPM) - PWV, Augmentation Index (AI) and Central Blood Pressure (CBP).

## Methods

### Study design and participants

This is a cross-sectional observational study. Eligible participants were those seen at the outpatient clinic of a university hospital, comprising a reference laboratory for vascular aging, where arterial stiffness is assessed by applanation tonometry with pulse wave velocity measurements (SphygmoCor®). This is an instrument that provides measurement of carotid-femoral pulse wave velocity (fc-PWV) in the femoral and carotid arteries by applanation tonometry. The system, validated and used for decades, is currently considered the gold standard non-invasive method for the acquisition of central hemodynamic measurements.^16^

Participants aged over 18 years were adopted as inclusion criteria. The exclusion criteria used were: absence of adequate techniques to verify peripheral BP,^17^ peripheral BP measurements not performed on digital, calibrated and validated devices; participation in other research protocols for less than one year according to ANVISA – Brazil regulations; chronic diseases in terminal stages; previous cardiovascular disease, including coronary artery disease (MI, angina, previous bypass surgery, or angioplasty) or stroke (ischemic stroke or TIA) for <6 months. The exclusion criteria for previous CVD presented were defined based on information obtained from the participants through direct interviews or evidence through complementary exams.

In the aforementioned outpatient unit, an average of 40 patients are seen per day, with an average of 200 patients per week, with central blood pressure measurements (CBPM) being performed in the indicated patients. The selection of participants was through an invitation to participate to those who met the inclusion and exclusion criteria, and acceptance by the patient. The sample size was 247 participants, according to the convenience of the field.

### Collected data

Data collection was performed at the time of routine patient care at the outpatient clinic in June and October 2019. Information such as gender, age and associated comorbidities was collected, evaluated by self-reference and through chronic use medications. Smokers were defined as those who consumed at least one cigarette a day.^18^

Weight (in kg) and height (in m) were also collected by calculating the body mass index (Quetelet’s formula);^19^ and waist circumference (in cm). All measurements were taken with individuals in the standing position using standards created for population health studies.^19,20^

The investigation of cardiac and vascular damage of the target organ was performed through Doppler echocardiography and carotid Doppler, using a TOSHIBA Xsario model device. The following parameters were analyzed: measurements of the interventricular septum and the posterior wall of the left ventricle, left ventricular mass index and left atrial volume, on Doppler echocardiography, and measurement of intima media thickness and presence of carotid plaques, on carotid Doppler

Microalbuminuria was defined as albumin excretion in the urine between 30 and 300 mg / 24 hours^21^ performed by means of a 24-hour urine collection or in the presence of the exam with less than 6 months of completion.

cf-PWV was measured with the CvMS Sphygmocor device (version 9 of the software, AtCor Medical) by applanation tonometry (PWVton) sequentially in the carotid and femoral arteries, blocked by an electrocardiogram signal recorded simultaneously.^22^

CAP measurement was performed by applanation tonometry, in a SphygmoCor® device, calibrated and clinically validated by the European Society of Hypertension (ESH) and the European Society Cardiology (ESC).^23^ The instrument consists of a tonometer (portable pressure sensor or transducer) coupled to a computer with dedicated software for data collection and analysis. When used in the radial artery, SphygmoCor® also obtains measurements related to central systolic (CSBP) and diastolic (CDBP) blood pressure, pulse pressure amplification (PPA), central pulse pressure (CPP) and Augmentation Index (AIx) by transfer function. When used on the carotid and femoral arteries, the system also calculates PWV.

### Sample size

Sample calculation was performed to estimate prevalence in a finite population of 1250 individuals, prevalence of systolic and central diastolic hypertension of 13.7%^24^, tolerable absolute error of 5%, and confidence coefficient of 97.5%, totaling a sample of 200 patients. 20% was added to guarantee losses due to deficiencies in the adequate completion of the questionnaire.

At the end of the collection, data from 247 participants were obtained and 7 were excluded, of which 5 due to lack of data on WC and the other two due to more than 30% of the questionnaire being incomplete, ending up with a sample of 240 patients.

### Statistical analysis

Categorical data are presented in absolute (n) and relative (%) frequencies. Numerical variables are presented as mean and standard deviation of the mean or median and interquartile range (25th-75th percentile). To verify the normality of data distribution, the Shapiro Wilk test was used. For comparison between groups, the Mann Whitney U test or the Kruskal Wallis test or the unpaired t-Student test or one-way ANOVA was used. For the analysis of correlation between variables, the Spearman or Pearson correlation coefficient was calculated.

Linear and logistic regression analysis was also performed, having as outcomes the cardiological exams and the determining variable WC classified as altered and normal; the other variables were used as adjustments to determine the confounding potential. The analyses were performed using STATA version 14.p and for all tests a significance level of 5% was considered.

### Ethical aspects

The research project was evaluated and approved by the Research Ethics Committee of the Hospital das Clínicas, Universidade Federal de Goiás (UFG), opinion number: 3,907,884, with the signature of the Free and Informed Consent Form (ICF) by all participants.

## Results

A total of 240 patients who attend at the outpatient clinic of a university hospital participated in the study; however, it was not possible to collect some information from all patients. The sample consisted mostly of males, middle-aged, overweight and mean WC above the upper normal limit for females.^17^ There was a high prevalence of smokers^25^ and more than a quarter of the population studied had CVD (Table 1). The CBPM showed a mean PWV close to the upper limit of normality for target organ damage (PWV > 10m/s)^17,23^ and a CAP above the upper limit of normality for the studied population^17^ (Table 1).

**Table 1:** Characterization of the sample and relationship with the waist circumference classification of patients treated at the outpatient clinic of a university hospital^17^

	Total sample n=240	Waist Circumference		p-value
		Normal 34(14,17%)	Altered 206(85,83%)	
Age, years, median [IQR] , n=240	60,25 [51,50-70,00]	54,00 [38,00-64,00]	63,00 [53,00-71,00]	0,004^1^
Gender, n(%), n=240				0,001^3^
Female	115(47,92)	7(20,59)	108(52,43)	
Male	125(52,08)	27(79,41)	98(47,57)	
Weight, kg, median [IQR] , n=237	75,10 [67,00;84,00]	64,80 [55,80-76,70]	75,75 [69,55-85,10]	<0,001^1^
Body mass index, kg/m^2^, median [IQR] , n=238	27,98 [25,40-31,86]	22,94 [21,47-25,40]	28,62 [26,23-32,46]	<0,001^1^
Nutritional Status, n(%), n=238				<0,001^3^
Eutrophic	72(30,25)	24(72,73)	48(23,41)	
Overweight	166(69,75)	9(27,27)	157(76,59)	
Waist circumference, cm, mean (SD) , n=240	99,95(11,59)	84,52(7,83)	102,49(10,04)	<0,001^2^
Smoking habit, n(%), n=240				0,023^4^
Non smoker	166(69,17)	24(70,59)	142(68,93)	
Smoker	37(15,42)	9(26,47)	28(13,59)	
Ex smoker	37(15,42)	1(2,94)	36(17,48)	
Chronic diseases, n(%), n=240				
Arterial hypertension	213(88,75)	28(82,35)	185(89,81)	0,203^3^
Dyslipidemia	179(74,58)	19(55,88)	160(77,67)	0,007^3^
Brain stroke	46(19,17)	5(14,71)	41(19,90)	0,639^4^
Diabetes Mellitus	95(39,58)	6(17,65)	89(43,20)	0,005^3^
Microalbuminuria, n(%), n=212	81(38,21)	7(26,92)	74(39,78)	0,282^4^
PSBP, mmHg, median [IQR], n=240	140,00 [128,00-154,00]	132,00 [122,00-154,00]	140,00 [129,00-154,00]	0,068^1^
PDBP,mmHg, median [IQR], n=240	77,50 [70,00-86,00]	77,50 [70-84]	77,50 [70,00-87,00]	0,464^1^
PWV, m/s, median [IQR], n=239	9,30 [7,90-11,30]	8,91 [7,28-10,32]	9,41 [8,00-11,40]	0,151^1^
PAC,mmHg, median [IQR], n=240	128,00 [116,00-141,00]	124,00 [110,00-138,00]	129,00 [117,00-141,00]	0,106^1^
AIx, %, mean (SD), n=240	29,55(12,46)	28,93(15,29)	29,65(11,96)	0,756^2^
Heart damage, n(%), n=183	98(53,55)	7(31,82)	91(56,52)	0,029^3^
Vascular damage, n(%), n=112	87(77,68)	9(69,23)	78(78,79)	0,482^4^

IQR: interquartile range; SD: standard deviation; n: absolute frequency; % relative frequency; CRP: C-reactive protein; PSBP: peripheral systolic blood pressure; PDBP: peripheral diastolic blood pressure; CSBP: central systolic blood pressure; AIx: Augmentation index; PWS: pulse wave speed.

1 Mann-Whitney;

2 Student’s t-test for independent samples;

3 Chi-square test;

4 Fisher’s exact test, all with 5% significance.

In females, who were overweight, smokers and ex-smokers, dyslipidemia, diabetes mellitus and heart damage, there was a higher frequency of altered WC. The medians of age, weight and BMI are higher in individuals with altered WC than in those with eutrophic WC (Table 1).

Lower AIx values and higher PWV values were observed in males. There were also higher PWV and CAP in patients with vascular damage, but no difference in these parameters was found in relation to smoking (Table 2).

**Table 2:** Differences in CBPM between sex, carotid Doppler and smoking habit

Variables	PWV		CAP		AIx	
	Median [IQ]	p-value	Median [IQ]	p-value	Mean (DP)	p-value
Gender		<0,001^1^		0,526^1^		0,010^3^
Female	8,88 [7,68-10,10]		127,00 [114,00-140,00]		31,70(12,01)	
Male	10,10 [8,30-12,15]		129,00 [117,00-141,00]		27,58(12,58)	
Altered Carotid Doppler		0,004^1^		0,037^1^		0,072^3^
No	8,90 [7,70;10,20]		127,00 [114,00;135,00]		26,32(13,66)	
Yes	10,36 [9,20-12,10]		133,00 [119,00-148,00]		30,93(10,38)	
Smoking Habit		0,219^2^		0,682^2^		0,437^4^
Non smoker	9,21 [7,84-11,36]		128,50 [116,00-146,00]		30,20(12,61)	
Smoker	9,11 [7,93-10,61]		128,00 [112,00-138,00]		27,45(9,94)	
Ex Smoker	10,10 [8,70-11,50]		126,00 [116,00-136,00]		28,73(13,97)	

n: absolute frequency of individuals; IQR: Interquartile-range; SD: standard deviation of the mean; PWV: Pulse wave velocity; CAP: Central arterial pressure; AIx: Augmentation index; p-value obtained by

1 Mann-Whitney test, or

2 Kruskal-Wallis test;

3 Student t-test;

4 Oneway ANOVA test, all with 5% level of significance.

There was an inverse and significant correlation between AIx and Weight, BMI and WC. There was also a direct and significant correlation between: PWV and age and WC; between CAP and age and WC; and between AIx and age (Table 3).

**Tabela 3:** Correlation between WC and CBPM

Variables	Correlation rho correlation coefficient (p-value)		
	PWV^1^	CAP^1^	AIx^2^
Age (years)	0,54 (<0,001)	0,20 (0,002)	0,33 (<0,001)
Weight (kg)	0,09 (0,192)	0,03 (0,671)	-0,31 (<0,001)
BMI (kg/m^2^)	0,11 (0,095)	0,11 (0,080)	-0,17 (0,009)
WC (cm)	0,33 (<0,001)	0,15 (0,020)	-0,10 (0,131)

CBPM: Central Blood Pressure Measurements; PWV: Pulse wave velocity; CAP: Central arterial pressure; AIx: Augmentation index; BMI: Body Mass Index; WC: Waist Circumference;.

1 Spearman’s correlation test or Pearson’s

2 with 5% significance level.

In the crude association analysis, there was a direct association between altered WC and PSBP. When using an age- and sex-adjusted model, there was an inverse association between altered WC and AIx. In another model adjusted for age, sex, smoking, nutritional status and comorbidities, altered WC was not associated with any of the parameters evaluated. Finally, in the model adjusted for determinant variables, the altered WC was only determinant for PSBP (Table 4).

**Tabela 4:** Association between Waist Circumference and Cardiological Exams

	Model 1			Model 2			Model 3			Model 4		
	Coef.	CI_95%_	p	Coef.	CI_95%_	p	Coef.	CI_95%_	p	Coef.	CI_95%_	p
PSBP*	0,02	0,00;0,05	0,042	0,02	-0,00;0,05	0,074	0,01	-0,02;0,04	0,495	0,03	0,00;0,06	0,030
PDBP*	0,01	-0,01;0,04	0,270	0,02	0,00;0,05	0,046	0,01	-0,02;0,03	0,594	0,00	-0,03;0,03	0,812
PWV*	0,03	-0,01;0,07	0,120	0,00	-0,03;0,04	0,792	-0,00	-0,04;0,04	0,867	-0,01	-0,05;0,03	0,705
CAP*	0,02	-0,00;0,04	0,084	0,01	-0,01;0,04	0,321	0,00	-0,03;0,03	0,905	0,02	-0,01;0,05	0,180
AIx	0,72	-3,83;5,27	0,756	-4,47	-8,90-0,04	0,048	-2,87	-7,91;2,14	0,259	2,65	-2,31;7,62	0,294
Heart damage	1,02	0,07;1,97	0,034	0,87	-0,21;1,96	0,115	0,09	-1,14;1,33	0,882	-0,08	-1,51;1,36	0,916
Altered ABPM	-0,50	-1,71;0,70	0,415	-0,24	-1,49;1,01	0,706	-0,93	-2,42;0,55	0,218	-1,55	-3,13;0,03	0,054
Vascular damage	0,50	-0,77;1,77	0,440	1,18	-0,32;2,69	0,124	0,30	-1,50;2,10	0,743	1,08	-0,87;3,03	0,279

Coef.: Coefficient of linear or logistic regression; 95%CI: 95% confidence interval; PWV: Pulse wave velocity; CAP: Central arterial pressure; AIx: Augmentation index; ABPM: Ambulatory blood pressure monitoring.

1 Linear regression analysis

2 Logistic regression analysis, using as determining variable waist circumference classified as normal vs altered and dependent variables on the cardiological exams.

* Used variable on the logarithmic scale due to the absence of normality. Model 1 – crude; Model 2 - adjusted for age and sex; Model 3 – adjusted for age, sex, smoking habit, nutritional status and comorbidities; Model 4 – adjusted by variables with p<0.20 in the binary analysis.

## Discussion

Excess abdominal obesity is associated with a variety of metabolic abnormalities and CVD.^8,26^ WC measurement is used as a surrogate indicator of visceral obesity to predict morbidity and mortality at the population level,^27–29^ in addition to being a low-cost and easy-to-use biomarker.^30^

Arterial stiffness is also related to CVD and atherosclerosis^31^ and has been a strong independent predictor of coronary events and cardiovascular mortality in several groups of patients.^12,32^ In this study, we examined the relationships between arterial stiffness measured by PWV and a specific cardiovascular risk factor: WC of 240 participants.

Univariate associations were therefore significant between WC and all components of CBPM, with the exception of AIx. The strongest correlation was observed between WC and fc-PWV (p<0.001), which is not surprising, considering that it is currently the gold standard method for assessing arterial stiffness.^33^

In our study, waist circumference was a significant determinant of arterial stiffness through fc-PWV. The association between increased body fat and high arterial stiffness was also found in other observational, cross-sectional and longitudinal studies, in agreement with our findings.^34–36^ Other mechanisms, such as those involving adipokines and endothelial regulation, may also explain this association,^35^ as well as the hypothesis of a negative impact on the health of large arteries caused by abdominal adiposity.^37^

Choi et al.^38^ showed no significant correlation between WC and PWV in their study,^38^ which can be explained by the fact that WC cannot distinguish between visceral and subcutaneous fat.^39^

Previous cross-sectional work demonstrates that the increase in abdominal obesity was associated with the decrease in AIx. This finding may have been due to a decrease in the transmural aortic pressure gradient and consequent reduction in the point of operational stiffness of the aorta or to subclinical left ventricular dysfunction that manifests as a lower degree of pressure increase for any given magnitude of reflection.^40,41^

Our study also showed no correlation between WC and AIx, but with a borderline p (p=0.06), suggesting that a likely larger sample could show a different result. When adjusted for age, there was also an inverse association between WC and AIx.

Shiva et al., however, who evaluated changes in AIx over a period of approximately 3 years, found that increasing waist circumference over time was associated with an increase in AIx. The direct prospective relationship between abdominal obesity and AIx suggests a progressive vascular dysfunction caused by obesity, resulting in a late increase in systolic pressure.^42^

Our study revealed that increasing WC had a positive association (p=0.002) with increasing CAP. A representative cohort of 2742 adults in Taiwan presented a multivariate analysis, which revealed that higher WC was independently associated with high CAP.^43^ The same association was also found in adolescents in the city of Salvador, Brazil.^44^ Previous studies showed results similar to ours,^24,45^ suggesting the benefit of measuring CAP as a better approach in the pathogenesis of cardiovascular diseases.

Our results must be interpreted within the context of the potential limitations of the study. First, most participants in our study had at least one CV risk factor among hypertension, dyslipidemia, diabetes, overweight, or cardiovascular comorbidities. Although these factors have been properly accounted for, our data may not be representative of an entire population.

Finally, it is estimated that the presence of a more expressive sample volume can improve the statistical power of the work and reinforce the benefits of the results presented.

## Conclusion

This study demonstrated a positive correlation between WC and arterial stiffness measured by fc-PWV and CAP, suggesting the evaluation of the effect of WC on vascular health as a method of aid in the early treatment of CVD and in the prevention of clinical outcomes. Therefore, future studies to determine the relationship between abdominal obesity and the risk of arterial stiffness may consider WC to estimate more accurately. Our study provides information that requires confirmation by a large-scale randomized clinical trial because the effects of observational studies may be overestimated.
